# Long-term efficacy and safety of lerodalcibep in heterozygous familial hypercholesterolaemia: the LIBerate-HeFH trial

**DOI:** 10.1093/eurheartj/ehad596

**Published:** 2023-08-28

**Authors:** Frederick Raal, Nyda Fourie, Russell Scott, Dirk Blom, Matthys De Vries Basson, Meral Kayikcioglu, Kate Caldwell, David Kallend, Evan Stein, LIBerate-HeFH Investigators, Traci Turner, Jean Bergeron, Artuela Caku, Avishay Elis, Ronen Durst, Zafer Yalim, Meral Kayikcioglu, Bahadir Kirilmaz, Atac Celik, Irfan Duzen, Abdurraham Oguzhan, Ibrahim Basarici, Frederick Raal, Dirk Blom, Marc Abelson, Matthys Basson, Lesley Burgess, Nyda Fourie, Eli Heggen, Emil Asprusten, Vimal Mehta, Raman Puri, Ashwani Mehta, Preeti Gupta, Milan Chag, Akshyaya Pradhan, Francisco Fuentes Jimenez, Fernando Civeira Murillo, Xavier Pinto Sala, Russell Scott

**Affiliations:** Carbohydrate and Lipid Metabolism Research Unit, Department of Medicine, Division of Endocrinology and Metabolism, Faculty of Health Sciences, University of the Witwatersrand, 7 York Road, Parktown, 2193 Johannesburg, Gauteng, South Africa; Iatros International, 20 Captain Proctor street , Brandwag, 9301 Bloemfontein, Free State, South Africa; New Zealand Clinical Research, 214 Antigua street, 8011 Christchurch, South Island, New Zealand; Division of Lipidology and Cape Heart Institute, Department of Medicine, University of Cape Town, 7701 Cape Town, Western Cape, South Africa; Tiervlei Trial Centre, Karl Bremer Hospital, Bellville, 7530 Cape Town, Western Cape, South Africa; Department of Cardiology, Ege University, 35000 Izmir, Turkey; LIB Therapeutics, Cincinnati, 45201 OH, USA; LIB Therapeutics, Cincinnati, 45201 OH, USA; LIB Therapeutics, Cincinnati, 45201 OH, USA

**Keywords:** Lerodalcibep, Low-density lipoprotein cholesterol, Familial hypercholesterolaemia

## Abstract

**Background and Aims:**

Lerodalcibep, a novel small recombinant fusion protein of a proprotein convertase subtilisin/kexin type 9 gene–binding domain (adnectin) and human serum albumin, demonstrated highly effective low-density lipoprotein cholesterol (LDL-C) reduction with monthly 300 mg in 1.2 mL subcutaneous dosing in Phase 2. In this global Phase 3 trial, the safety and efficacy of lerodalcibep were evaluated in heterozygous familial hypercholesterolaemia patients requiring additional LDL-C lowering.

**Methods:**

Patients were randomized 2:1 to monthly subcutaneous injections of either lerodalcibep 300 mg or placebo for 24 weeks. The primary efficacy endpoints were the per cent change from baseline in LDL-C at Week 24 and the mean of Weeks 22 and 24.

**Results:**

In 478 randomized subjects [mean age (range); 53 (18–80) years, 51.7% female, mean (SD) baseline LDL-C 3.88 (1.66) mmol/L], lerodalcibep reduced LDL-C, compared with placebo by an absolute amount of 2.08 (0.11) mmol/L [LS mean (SE); 95% confidence interval −2.30 to −1.87] with a percentage difference of −58.61 (3.25)% at Week 24 and by 2.28 (0.10) mmol/L (95% confidence interval −2.47 to −2.09) with a percentage difference of −65.0 (2.87)% at the mean of Weeks 22 and 24 (*P* < .0001 for all). With lerodalcibep, 68% of subjects achieved both a reduction in LDL-C ≥ 50% and the recommended European Society of Cardiology LDL-C targets during the study. Except for mild injection site reactions, treatment-emergent adverse events were similar between lerodalcibep and placebo.

**Conclusions:**

Lerodalcibep, a novel anti-proprotein convertase subtilisin/kexin type 9 gene small binding protein dosed monthly as an alternative to monoclonal antibodies, significantly reduced LDL-C in subjects with heterozygous familial hypercholesterolaemia with a safety profile similar to placebo.

## Structured Graphical Abstract

**Structured Graphical Abstract:**
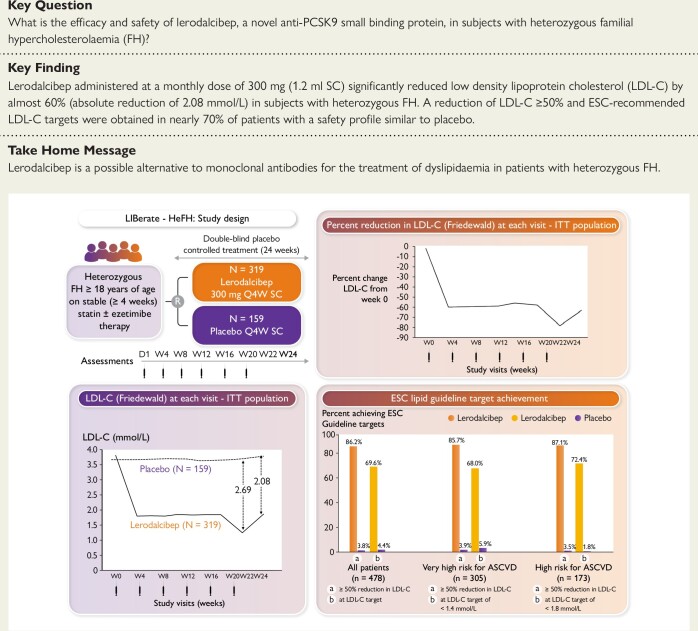
Lerodalcibep, a novel anti-proprotein convertase subtilisin/kexin type 9 gene small binding protein for the management of heterozygous familial hypercholesterolaemia. ASCVD, atherosclerotic cardiovascular disease; ESC, European Society of Cardiology; FH, familial hypercholesterolaemia; ITT, intention to treat; LDL-C, low-density lipoprotein cholesterol; SC, subcutaneous.

**See the editorial comment for this article ‘Third generation PCSK9-inhibitors’, by U. Laufs *et al*., https://doi.org/10.1093/eurheartj/ehad566.**

## Introduction

Heterozygous familial hypercholesterolaemia (HeFH), an autosomal semi-dominant genetic disorder that affects approximately 1 in 300 persons or over 30 million people worldwide, is characterized by elevated low-density lipoprotein cholesterol (LDL-C) levels from birth and, if untreated, is associated with premature cardiovascular morbidity and mortality from accelerated atherosclerotic cardiovascular disease (ASCVD).^1,2^ The condition results from dysfunctional variants in genes responsible for the clearance of LDL-C. This is mainly due to loss-of-function variants in the low-density lipoprotein receptor gene (LDLR) and less commonly the apolipoprotein B gene (APOB) or gain-of-function variants in the proprotein convertase subtilisin/kexin type 9 gene (PCSK9).^3^ As untreated HeFH is frequently associated with the development of ASCVD in the fourth or fifth decade, it is imperative to initiate effective LDL-C reduction to achieve guideline-directed goals as early as possible.^1,4^ In those with pre-existing ASCVD or multiple ASCVD risk factors, the consensus target is an absolute LDL-C of <1.4 mmol/L, plus at least a 50% reduction in LDL-C from baseline. For HeFH patients without ASCVD or major risk factors, the target is a LDL-C of <1.8 mmol/L together with a reduction of ≥50% from baseline.^5,6^ Pharmacological management of familial hypercholesterolaemia (FH) includes high-intensity statins with or without ezetimibe and, in those who fail to achieve LDL-C targets, additional therapies inhibiting or reducing circulating PCSK9.^7^ Monoclonal antibodies (mAbs), such as evolocumab and alirocumab, prevent PCSK9 from binding to the LDLR, enabling rapid recycling of the LDLR, and significantly reduce LDL-C due to enhanced clearance even in subjects with HeFH and have been shown to be safe and effective in the long term.^8–10^ These systemic agents that work only in the circulation offer distinct advantages over other pharmacologic interventions that utilize small drug molecules, including a reduced risk for drug–drug interactions because they are not metabolized by the liver or cleared by the kidney and do not enter hepatic or muscle cells, or interact with cytochrome P450 or other transport proteins, or reduce intracellular cholesterol synthesis or its downstream products such as ubiquinone.^11^ Furthermore, mAbs have a high specificity for target antigens, achieving a high potency with less frequent, but subcutaneous (SC), dosing, and do not penetrate the central nervous system because of their size.^11^

Lerodalcibep (LIB003), a novel small recombinant fusion protein of a PCSK9-binding domain (adnectin) and human serum albumin, demonstrated highly effective LDL-C reduction with monthly 300 mg in 1.2 mL SC dosing in Phase 2.^12,13^ In this large global Phase 3 trial, we evaluated the safety and efficacy of lerodalcibep in HeFH patients, on maximally tolerated statins and other oral lipid-lowering agents, requiring additional LDL-C lowering.

## Methods

### Study design and population

Liberate-HeFH was a Phase 3, global, placebo-controlled, randomized, double-blind study in subjects with HeFH conducted in nine countries across 30 sites. The protocol and informed consent were approved by the Institutional Review Board/Independent Ethics Committee and local regulators at all clinical sites before enrolment of any subjects. The diagnosis of FH was based on genetic confirmation or established phenotypic criteria using either the Simon Broome or Dutch Lipid Network Criteria.^14,15^ In addition, eligible subjects were required to have a LDL-C of ≥1.8 mmol/L if they had established ASCVD or ≥2.6 mmol/L in the absence of ASCVD, and a triglyceride level of ≤4.5 mmol/L, while on stable maximally tolerated statin and other background lipid-lowering oral drug therapies. Patients known to have homozygous FH were excluded, but there was no upper cut-off level for LDL-C. Patients with documented intolerance to statins could also participate. Subjects were excluded if they had been receiving treatment with mAbs directed towards PCSK9 within 8 weeks of screening or within the past year if treated with the small interfering RNA (siRNA), inclisiran. Detailed inclusion and exclusion criteria are provided in Supplementary data online, *Appendix S1*.

### Trial procedures

Patients who met eligibility criteria were randomized 2:1 to monthly SC injections of either lerodalcibep 300 mg or placebo for 24 weeks. Following randomization and dosing on Day 1, patients were seen in the clinic every 4 weeks (≤31 days) for 20 weeks and then at 2-week intervals for Weeks 22 and 24. In addition to a basic lipid profile and laboratory safety parameters at each visit, LDL-C was also measured by preparative ultracentrifugation. In addition, apolipoprotein B and lipoprotein(a) [Lp(a)], free (unbound) PCSK9, lerodalcibep levels, and immunogenicity were measured at specified visits during the study. Treatment and efficacy were blinded to investigators, study staff, participants, and sponsor personnel involved in the study.

### Outcomes

The primary efficacy endpoints, based on the intention-to-treat population, were the per cent change from baseline in LDL-C at Week 24 and the mean of Weeks 22 and 24. Secondary efficacy endpoints include changes in apolipoprotein B, Lp(a), and achievement of European Society of Cardiology (ESC)-recommended LDL-C targets.

### Safety reports

We recorded adverse events, clinical laboratory values, and vital signs at all visits through to the end-of-trial visit. Electrocardiograms were performed at screening, Day 1 and Week 24. Investigators classified adverse events using a standard Medical Dictionary for Regulatory Activities (MedDRA version 23.1) according to organ class and as mild, moderate, or severe. Adverse events at the injection site were evaluated using pre-specified methods and terms. Cardiovascular events were collected and were adjudicated by an independent committee. Serum was collected for immunogenicity [anti-drug antibodies (ADAs)] at all visits, with ADAs initially analysed for patients receiving lerodalcibep on Day 1 and Week 24. Patients confirmed positive for lerodalcibep antibodies at Week 24 were tested for neutralizing antibodies (NAbs). Serum pharmacokinetic drug levels and free PCSK9 were measured at Weeks 12 and 24 in all subjects in the lerodalcibep-treated group and in 14% of those in placebo in order to maintain blinding by the laboratory. In addition to safety monitoring by the medical monitor and clinical staff, a formally constituted independent committee monitored safety, including cardiovascular events.

### Statistical analysis

The co-primary efficacy endpoints were to assess per cent change from baseline compared with placebo in LDL-C level calculated by Friedewald formula at Week 24 and at the mean of Weeks 22 and 24.^16^ Per cent change from baseline in LDL-C was analysed with analysis of covariance model with treatment and ASCVD status as factors and the baseline value as a covariate. Missing data were imputed using a washout model. Under this model, data that are missing after a subject discontinues the trial are considered missing not at random (MNAR). For the co-primary endpoints, the family-wise type I error rate was controlled at a significance level of alpha = .05 using a fallback procedure. Under this procedure, the per cent change from baseline in LDL-C was first compared with placebo at Week 24 using a two-sided significance level of alpha = .05. If the null hypothesis was rejected and superiority of lerodalcibep over placebo was claimed, testing would proceed with the second primary endpoint of the per cent change from baseline in LDL-C level at the mean of Weeks 22 and 24 using a two-sided significance level of alpha = .05.

Other efficacy variables were analysed similarly. The percentage of patients achieving a ≥50% reduction in LDL-C and in those with ASCVD or at very high risk for ASCVD, an LDL-C of <1.4 mmol/L, and the percentage of patients without ASCVD but at high risk for ASCVD achieving a LDL-C of <1.8 mmol/L were analysed using a logistic regression model with treatment and ASCVD status as factors and baseline value as a covariate. A sensitivity analysis of the co-primary endpoints was also performed by application of a mixed-effect model repeat measurement and rank analysis of covariance (Quade test) for patients who adhered to the scheduled study per-protocol drug administration with visits <31 days and who did not have any missing data for the co-primary endpoints.

## Results

### Baseline characteristics

The trial was conducted between 3 May 2021 (first patient screened) and 15 May 2023 (last patient last visit), with 570 patients screened and 478 randomized. The mean age (range) for all patients was 53 (18–80) years; 247 (51.7%) were female and the majority [*n* = 416 (87%)] were White with 13% South Asian, multi-racial, or Black/African-American. Pre-existing coronary heart disease was present in 157 patients (47.6%) and diabetes in 49 patients (10.3%). Mean baseline (± SD) LDL-C was 3.88 (1.66) mmol/L, despite 88.3% of subjects being on statins, with 66.7% on high-intensity statins and nearly half (48.7%) also on ezetimibe (*Table 1*). A total of 319 patients were randomized to lerodalcibep and 159 to placebo. Of these, 306 (95.9%) patients in the lerodalcibep group and 155 (97.5%) in the placebo group completed the last dose of study drug (see Supplementary data online, *Figure S1*).

**Table 1:** Demographics and clinical characteristics of the study cohort at baseline (intention-to-treat population)

Characteristic	Placebo (*n* = 159)	Lerodalcibep (*n* = 319)
Age, years (range)	53.8 (18–79)	52.5 (19–80)
Female sex, no. (%)	83 (52.2)	164 (51.4)
Race White no. (%)	143 (89.9)	273(85.6)
Black/multi-racial no. (%)	12 (7.5)	25 (7.8)
South Asian no. (%)	4 (2.5)	21 (6.6)
Body mass index, kg/m^2^ mean(SD)	28.79 (5.14)	28.83 (5.05)
Diagnosis of HeFH no. (%)	159 (100)	319 (100)
On clinical criteria no. (%)	103 (64.8)	186 (58.3)
On genotyping no. (%)	56 (35.2)	133 (41.7)
Diabetes mellitus no. (%)^a^	18 (11.3)	31 (9.7)
Documented ASCVD no. (%)	49 (45.0)	108 (48.9)
Very-high risk for ASCVD no. (%)	109 (68.6)	221 (69.3)
High risk for ASCVD no. (%)	50 (31.4)	98 (30.7)
**Lipid modifying therapy, no. (%)**		
Statin; any dose	141 (88.7)	281 (88.1)
High-intensity statin	101 (63.5)	218 (68.3)
Not high-intensity statin	40 (25.2)	63 (19.7)
Ezetimibe	78 (49.1)	155 (48.6)
**Lipid measures at baseline**		
LDL-cholesterol (calculated), mmol/L; mean(SD)	3.79 (1.47)	3.93 (1.74)
LDL-cholesterol (UC), mmol/L; mean(SD)	3.74 (1.39)	3.86 (1.66)
Total cholesterol, mmol/L; mean(SD)	5.76 (1.60)	5.94 (1.84)
Non HDL-cholesterol, mmol/L mean(SD)	4.44 (1.61)	4.63 (1.87)
HDL-cholesterol, mmol/L; mean(SD)	1.33 (0.39)	1.31 (0.41)
Apolipoprotein B, mg/dL; mean(SD)	119.9 (37.68)	123.3 (42.87)
Lipoprotein(a), nmol/L; median (IQR)	46.0 (158)	57.0 (148)
Triglycerides, mmol/L; median (IQR)	1.17 (0.82)	1.23 (0.93)
PCSK9, ng/mL; mean (SD)		341 (120)^b^

ASCVD, atherosclerotic cardiovascular disease; HeFH, heterozygous familial hypercholesterolemia; UC, preparative ultracentrifugation.

Patient reported.

*n* = measured in lerodalcibep treated patients only.

### Primary outcome

**Figure 1:**
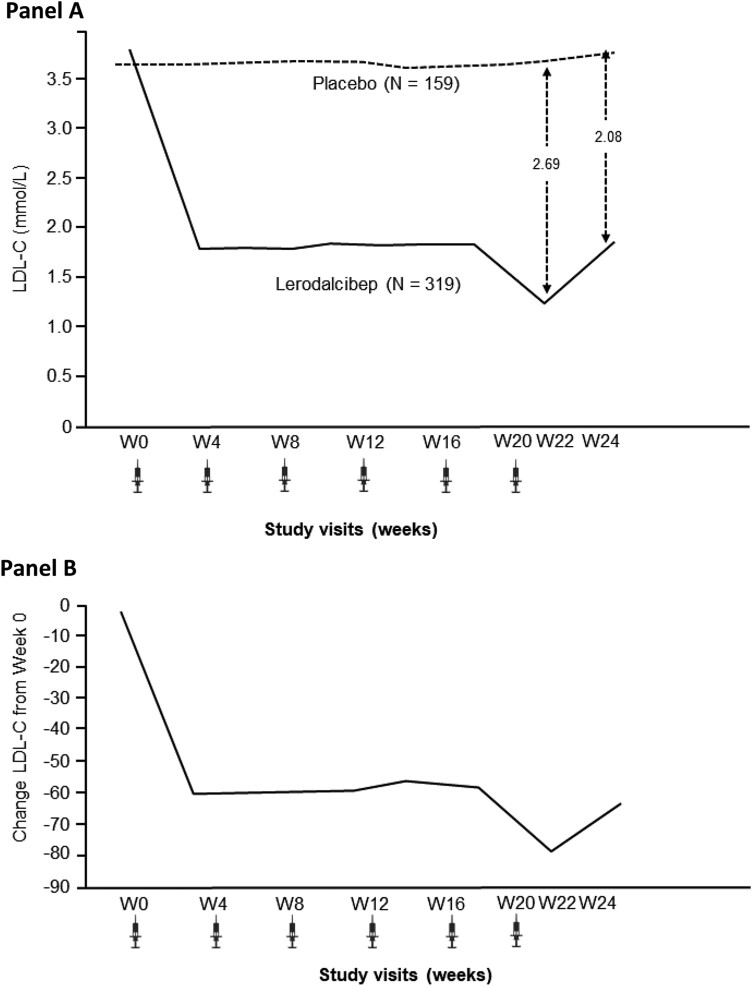
Low-density lipoprotein cholesterol (Friedewald) at each visit—intention-to-treat population; absolute reduction in mmol/L (*A*) and placebo adjusted per cent reduction (*B*)

Lerodalcibep reduced LDL-C from baseline by an absolute amount of 2.08 (0.11) mmol/L [LS mean (SE); 95% confidence interval (CI) −2.30 to −1.87] with a placebo-adjusted −58.61 (3.25)% reduction at Week 24 (*P* < .0001 for both). For the co-primary endpoint, the mean of Weeks 22 and 24, lerodalcibep reduced LDL-C by 2.28 (0.10) mmol/L (95% CI −2.47 to −2.09) and −65.0 (2.87)% compared with placebo (*P* < .0001 for both). The absolute and percentage reductions in LDL-C with lerodalcibep compared with placebo during the study are shown in *Figure 1*.

### Secondary outcomes

**Figure 2:**
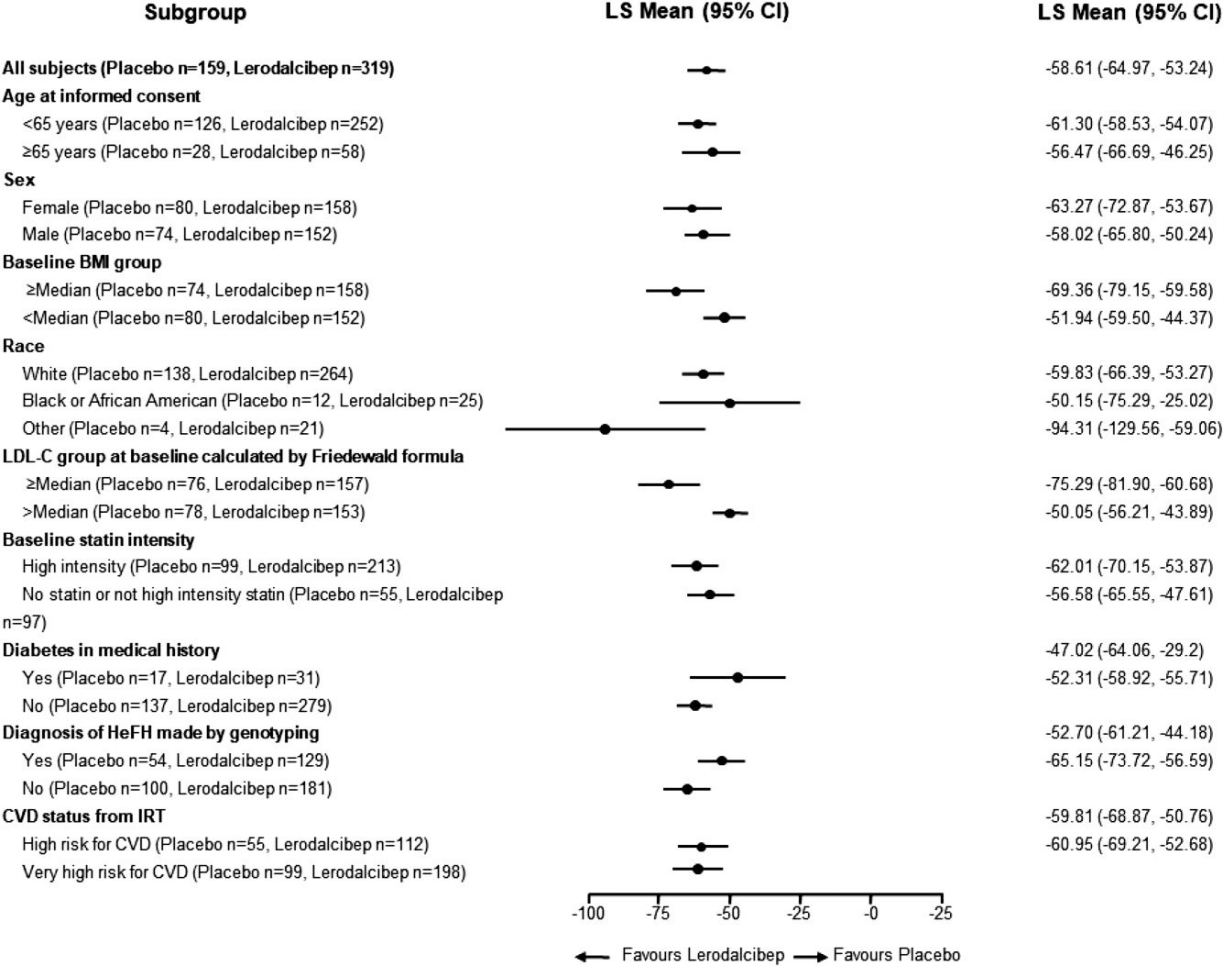
Forest plot showing low-density lipoprotein cholesterol reduction with lerodalcibep in different subgroups. BMI, body mass index; IRT, interactive response technology

**Figure 3:**
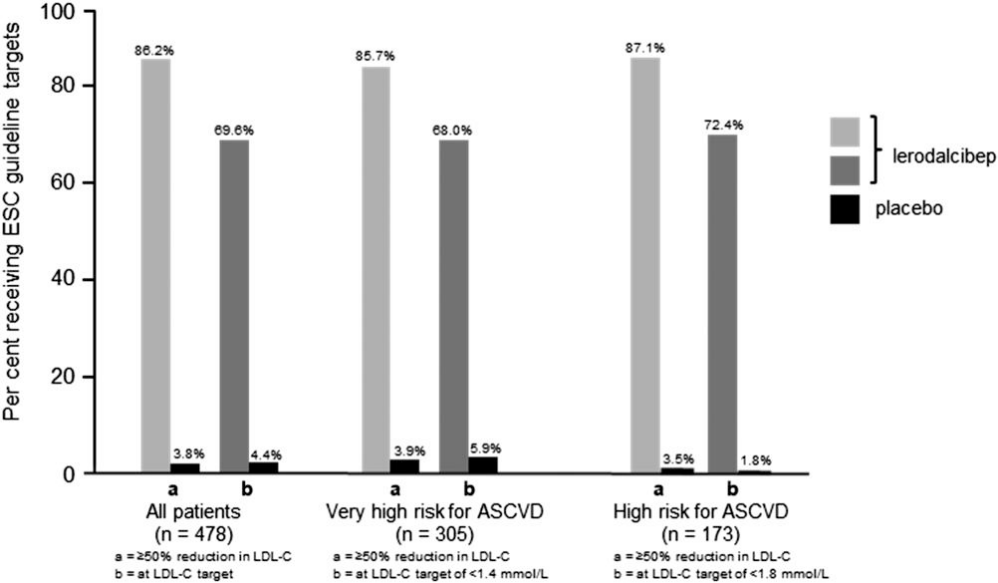
Percentage of patients achieving a ≥50% reduction in low-density lipoprotein cholesterol and European Society of Cardiology–recommended low-density lipoprotein cholesterol targets with lerodalcibep compared with placebo during the study

The effect of lerodalcibep on LDL-C reduction was consistent across a range of subgroups (*Figure 2*). Importantly, the reduction in LDL-C was independent of body mass index, sex, or background oral lipid-lowering therapy. A reduction in LDL-C of ≥50% was achieved in 275 subjects (86.2%) with lerodalcibep, and ESC LDL-C targets were achieved in 222 (69.6%) subjects (*Figure 3*).

In the per-protocol analysis, 84% of subjects in the lerodalcibep group and 82% on placebo adhered to the scheduled study per-protocol drug administration with visits <31 days and did not have any missing data for the co-primary endpoints of LDL-C at Weeks 22 and 24. The LS mean (SE) placebo-adjusted absolute reduction in LDL-C was 2.25 mmol/L or −63.62 (3.27)% (95% CI −70.05% to −57.19%; *P* < .0001) at Week 24, and at Week 22/24, endpoint was LS mean (SE) 2.45 mmol/L or −70.23 (2.83)% (95% CI −75.79% to −64.67%; *P* < .0001) (see Supplementary data online, *Table S2* ).

The majority (68%) of subjects on lerodalcibep achieved both a reduction in LDL-C of ≥50% and the ESC-recommended LDL-C target values vs. only 1.9% on placebo [odds ratio (95% CI) 220.23 (65.77–737.45); *P* < .0001] during the study. Of those with ASCVD, or at very high risk for ASCVD (*n* = 305), 85.7% achieved a reduction in LDL-C of ≥50% with 68% achieving the recommended LDL-C target of <1.4 mmol/L with lerodalcibep, compared with only 3.9% and 5.9%, respectively, with placebo. For the remainder at high risk for ASCVD (*n* = 173), 87% achieved a reduction in LDL-C of ≥50% and 72.4% achieved the LDL-C target of <1.8 mmol/L with lerodalcibep compared with only 3.5% and 1.8% respectively with placebo. The percentages who achieved a ≥50% reduction in LDL-C and who reached ESC-recommended LDL-C target values were similar using the means of Week 22 and Week 24 visits (see Supplementary data online, *Table S3*). Lerodalcibep reduced free PCSK9 levels from baseline by a median of 93.4% and 94.3% at Weeks 12 and 24, respectively (*P* < .0001 for both) compared with an increase of 0.6% at Week 12 and a decrease of 1.2% at Week 24 on placebo. Lerodalcibep reduced mean apolipoprotein B by −52.2 (2.65) mg/dL [LS mean (SE); 95% CI −57.4 to −47.0; *P* < .0001], a 45.6 (2.26)% (95% CI −50.02 to −41.16; *P* < .0001) reduction compared with placebo, and reduced median Lp(a) by 24.4% (interquartile range 37–53; *P* < .0001). Consistent with the reductions in LDL-C, total cholesterol and non–high-density lipoprotein cholesterol decreased significantly compared with placebo, as did triglycerides (*P* < .0001 for all) while a moderate but statistically significant (*P* < .01) increase, in high-density lipoprotein cholesterol, was seen compared with placebo (*Table 2*). The percentage change in LDL-C levels from baseline to Week 24 with lerodalcibep and placebo in individual patients is shown as a waterfall plot in Supplementary data online, *Figure S2*.

**Table 2:** Changes in lipid and free proprotein convertase subtilisin/kexin type 9 gene parameters at week 24 (intention-to-treat population)

Parameter	Lerodalcibep (*n* = 319)	Placebo (*n* = 159)	Placebo adjusted	*P* value
LDL-C (calculated)^a^	−50.5%	+8.1%	−58.6%	<.0001
LDL-C (UC)	−47.9%	+8.1%	−56.0%	<.0001
Total cholesterol	−32.8%	+4.9%	−37.7%	<.0001
Apolipoprotein B	−38.9%	+6.7%	−45.6%	<.0001
Non-HDL-C	−45.2%	+7.5%	−52.6%	<.0001
VLDL cholesterol	−16.2%	+9.4%	−25.6%	<.0001
Triglyceride (median)	−10.4%	+2.2%	−12.2%	<.0001
Lp(a) (median)	−24.4%	−1.4%	−23.9%	<.0001
HDL-C	+8.2%	+3.3%	+4.8%	<.01
Free PCSK9 (median)	−94.3%	-	-	<.0001^b^

Calculated using the Friedewald formula (Haase and Goldberg^15^).

*P* values evaluate the percentage change from baseline within the lerodalcibep group. All other *P* values are for the comparison with placebo.

### Safety and adverse events

The incidence of treatment-emergent adverse events is shown in *Table 3*. Adverse events that occurred during the study period, regardless of causality, were reported in 230/318 (72.3%) receiving lerodalcibep and in 109/159 (68.6%) receiving placebo. The majority of events [221/230 (96%) in the lerodalcibep patients and 101/109 (93%) placebo, respectively] were reported as mild to moderate. There was a lower number of serious adverse on lerodalcibep (9/318; 2.8%) compared with placebo (9/159; 5.7%). Adverse event rates were also similar between lerodalcibep and placebo when assessed according to system–organ class. Other safety parameters including laboratory abnormalities were rare and occurred at a similar incidence in both treatment groups. Mean fasting blood glucose and glycated haemoglobin levels were unchanged from baseline to Week 24 in both the lerodalcibep and placebo groups. There was also no difference in the incidence of adverse events related to glycaemic control between the groups. There were seven adjudicated events, three (1.9%) in the placebo group (one death, one ischaemic stroke, and one cardiac event), and four (1.3%) with lerodalcibep (all cardiac events). None were considered to be study drug related.

**Table 3:** Adverse events and key safety laboratory findings

	Placebo (*n* = 159)	Lerodalcibep (*n* = 318)	Risk difference (95% CI)
**Adverse events, *n* (%)**			
Patients with at least one adverse event	109 (68.6)	230 (72.3)	3.8 (−5.9 to 13.4)
Patients with at least one event leading to withdrawal of study drug	3 (1.9)	5 (1.6)	
**Serious adverse events**			
Patients with at least one serious adverse Event	9 (5.7)	9 (2.8)	
Death	1 (0.6)	0	
Cardiovascular deaths	1 (0.6)	0	
Cancer deaths	0	0	
**Other cardiovascular adverse events**			
Pre-specified exploratory cardiovascular events^a^	4 (2.5)	4 (1.3)	
Fatal and non-fatal myocardial infarction	0	0	
Fatal and non-fatal stroke	0	0	
**Frequent adverse events** ^b^			
COVID-19	17 (10.7)	35 (11.0)	0.3 (−9.3 to 10.0)
Upper respiratory tract infection	11 (6.9)	18 (5.7)	−1.3 (−10.9 to 8.4)
Arthralgia	12 (7.5)	13 (4.1)	−3.5 (−13.1 to 6.2)
Nasopharyngitis	3 (1.9%)	16 (5.0)	3.1 (−6.5 to 12.8)
Urinary tract infection	8 (5.0)	10 (3.1)	−1.9 (−11.5 to 7.8)
**Laboratory results, *n* (%)**			
Liver function ALT or AST > 3× ULN	1 (0.6)	0	
AST or ALT > 5× ULN	0	0	
Muscle CK > 5× ULN	2 (1.3)	3 (0.9)	
Haematology platelet count < 75 × 10^9^/L	0	0	

Safety population includes all patients who received at least one dose of study medication and adverse events over the study period of 24 weeks.

ALT, alanine transaminase; AST, aspartate transaminase; ULN, upper limit of normal; CK, creatine kinase; RR, relative risk; CI, confidence interval.

MedDRA-defined cardiovascular basket of non-adjudicated terms including those classified within cardiac death and any signs or symptoms of cardiac arrest, non-fatal myocardial infarction, and/or stroke.

Defined as occurring with a frequency of 5% or more in either treatment group.

As the study was conducted during the COVID-19 pandemic, acute COVID-19 infections were reported in 35/318 (11.0%) of patients receiving lerodalcibep and in 17/159 (10.7%) of those on placebo.

Other treatment-related adverse events included injection site reaction (ISR) with lerodalcibep in 10.1% (32/318) of subjects compared with 1.3% (2/159) with placebo (see Supplementary data online, *Table S1*). The number of total ISR events was 57 (3.1%) of the 1853 doses of lerodalcibep administered compared with 2 (0.2%) of the 934 doses with placebo. All ISRs were graded as mild or moderate; none were considered severe or persistent and did not lead to higher withdrawal from treatment than placebo (*Table 3*). In addition, despite a higher incidence of ISRs with lerodalcibep, the percentage of patients who discontinued the study was lower with lerodalcibep (1.6%) compared with placebo (2.5%).

Low levels of transient and sporadic ADAs were detected, and 11 patients (3.4%) had *in vitro* NAbs. None of the NAbs were associated with *in vivo* effects, specifically ISRs or any impairment in PCSK9 or LDL-C reduction efficacy.

## Discussion

Heterozygous FH is one of the most common inherited conditions worldwide, which, if untreated or undertreated, may confer a 10-fold or greater risk of premature ASCVD as compared with the general population.^17,18^ The major driver of ASCVD risk in patients with FH is the life-long cumulative exposure to elevated LDL-cholesterol particularly when there is a delay in initiation of effective LDL-C-lowering therapy.^19^ In the last decade mAbs to PCSK9, evolocumab and alirocumab were the first novel and highly effective agents to be added to the standard therapy, since statins and ezetimibe, for the treatment of FH.^20^ In HeFH patients on stable lipid-lowering therapy, the addition of evolocumab 140 mg Q2W or 420 mg Q4W significantly reduced LDL-C levels over 12 weeks by up to 60% compared with placebo.^8^ Alirocumab, at doses of 75 mg Q2W, 150 mg Q2W, or 300 mg Q2W, in HeFH patients lowered LDL-C by 39.1%–57.9% at 12 weeks compared with placebo.^9,21^ However, these therapies need to be administered subcutaneously every 2 weeks, or with multiple injections or a 9-min slow infusion device, if higher doses are administered monthly.

An alternative to binding or inhibiting circulating PCSK9 has been to inhibit the synthesis of hepatic PCSK9 production with a siRNA, inclisiran, and reduce PCSK9 entering the circulation. In the ORION-9 study, a Phase 3 trial in adults with HeFH, with an LDL-C level of >2.6 mmol/L despite oral lipid-lowering therapy, the administration of 300 mg inclisiran at Day 1, Day 90, and then every 6 months resulted in a placebo-adjusted peak reduction of 48% in LDL-C at Day 510, 60-day post-prior dose, with a time-averaged reduction in the LDL-C between Day 90 and Day 540 of 44% and trough reduction of 40%, at Day 270 prior to the next dose.^22^ The reduction in LDL-C is less, and between dose variability greater, than that seen with mAb therapy, as inclisiran reduces hepatic PCSK9 synthesis reducing circulating PCSK9 by approximately 80% at peak and 60% at trough, while not effecting renal, gut, and other sites of PCSK9 production, which contribute to circulating PCSK9.^23–25^

While oral PCSK9 inhibitors are in early development and appear promising, daily administration is necessary, and, as food significantly reduces absorption, patients are required to fast and not consume food for at least 30-min post-dose. It also remains to be shown if tolerability and efficacy during longer-term treatment with oral PCSK9 inhibitors are sustained especially in patients on multiple medications with established ASCVD or with multiple co-morbidities at very high risk for ASCVD. In addition, as ASCVD risk reduction is driven by the absolute reductions in LDL-C, it will be important to determine if LDL-C efficacy in patients with more severe LDL-C elevations, such as those with FH, on maximally tolerated statins and other oral lipid-lowering agents, will be comparable with that achieved with injectables.^19^ In this study, lerodalcibep, a novel small binding protein developed on an alternative platform to mAbs but with the same mechanism of action by binding to PCSK9 in the circulation at a dose of 300 mg in a volume of only 1.2 mL administered monthly, provided sustained LDL-C efficacy and free PCSK9 suppression and was as effective as evolocumab 140 mg administered every 2 weeks or with monthly 420 mg dosing delivered as either a 3.5 mL 9-min slow infusion or three separate injections. The mean reduction in LDL-C of over 2.69 mmol/L at peak 2-week post-dosing at Week 22, and over 2.08 mmol/L at trough 4-week post-dosing at Week 24, allowed the majority of HeFH patients, even those with high LDL-C levels despite treatment with high-intensity statin and ezetimibe, to achieve currently recommended ESC LDL-C targets. The 84% of subjects who maintained adherence to the protocol-defined monthly dosing visits achieved mean reductions in LDL-C compared with placebo at the 24-week trough of 64% with just over a 70% reduction at peak during Week 22 through to Week 24. In addition, lerodalcibep lowered apolipoprotein B by almost 50%. Lipoprotein(a) was reduced by 24% consistent with reduction seen with other PCSK9-directed therapies. As elevated Lp(a) is an independent risk factor for ASCVD disease, even in HeFH, the significant reductions in Lp(a) may be an additional benefit of PCSK9 inhibition therapy.^26^

While this study was of a relatively short 24-week duration, it is longer than many of the prior Phase 2 and 3 trials in HeFH with mAbs. It is also the largest trial in terms of most ethnically diverse subjects and actively treated patients with HeFH with a PCSK9 inhibitor. An additional strength of the study was that the trial did not have an upper limit for LDL-C at study entry and included patients with on-treatment LDL-C levels over 10 mmol/L providing a robust assessment of lerodalcibep and PCSK9 inhibition effectiveness in more severe HeFH patients.

In summary, this large global trial with a novel small binding protein to PCSK9 in subjects with HeFH demonstrated that the addition of lerodalcibep 300 mg monthly to existing maximally tolerated statin, ezetimibe, and other lipid-lowering therapies achieved additional LDL-C reductions of approximately 60% and allowed nearly 70% of patients to achieve both a reduction in LDL-C of ≥50% and the 2019 ESC-recommended LDL-C targets, with a safety profile similar to placebo (*Structured Graphical Abstract*). These results support the use of lerodalcibep for the management of HeFH.

## Supplementary Material

ehad596_Supplementary_Data

## Data Availability

Researchers may request access to study documents (including the clinical study report, study protocol with any amendments, blank case report form, statistical analysis plan) that support the methods and findings reported in this manuscript. Individual anonymized participant data will only be made available once the indication has been approved by a regulatory body, if there is legal authority to share the data and there is not a reasonable likelihood of participant re-identification.

## Pre-registered Clinical Trial Number

EudraCT: 2020-004390-44; Cliniclatrials.gov NCT04797104.
