# Supplementary material for: Long-term efficacy and safety of lerodalcibep in heterozygous familial hypercholesterolaemia: the LIBerate-HeFH trial
Source: Eur Heart J. 2023 Aug 28;44(40):4272–80. doi: 10.1093/eurheartj/ehad596 (PMC10590131; doi:10.1093/eurheartj/ehad596)
Supplement: ehad596_Supplementary_Data [file ehad596_supplementary_data.docx]

**Supplementary Appendix**

This appendix has been provided by the authors to give readers additional information about their work.

**Supplement to: Long-term efficacy and safety of Lerodalcibep in heterozygous familial hypercholesterolaemia: the LIBerate-HeFH trial**

Frederick Raal,^1^ Nyda Fourie,^2^ Russell Scott,^3^ Dirk Blom,^4^ Matthys De Vries Basson,^5^ Meral Kayikcioglu,^6^ Kate Caldwell,^7^ David Kallend,^7^ Evan Stein^7^ for the LIBerate-HeFH Investigators

From ^1^Carbohydrate and Lipid Metabolism Research Unit, Department of Medicine, Witwatersrand University, Johannesburg, South Africa; ^2^Iatros International, Bloemfontein. South Africa; ^3^New Zealand Clinical Research, Christchurch. New Zealand; ^4^Division of Lipidology and Cape Heart Institute, Department of Medicine, University of Cape Town, Cape Town, South Africa; ^5^Tiervlei Trial Centre, Karl Bremer Hospital, Bellville, Cape Town. South Africa South Africa; ^6^Department of Cardiology, Ege University, Izmir, Turkey; ^7^LIB Therapeutics, Cincinnati, OH, USA

# Study Oversight

| **STUDY DESIGN AND DURATION:**  This was a randomized, double-blind, placebo-controlled, Phase 3 study of 24 weeks duration. Approximately 600 males and females aged ≥18 years who fulfilled the inclusion and exclusion criteria were to be enrolled at up to 60 sites in the United States, Canada, Central and South America, Europe, South Africa, Asia, Australasia, and the Middle East. Patients were randomized in a 2:1 ratio to lerodalcibep (400 patients) or placebo (200 patients) administered SC Q4W (≤31 days). The study consisted of a Screening Period and a Treatment Period. The total study duration was up to 32 weeks which included a potential up to 8-week washout period, and 24 weeks of study drug treatment.  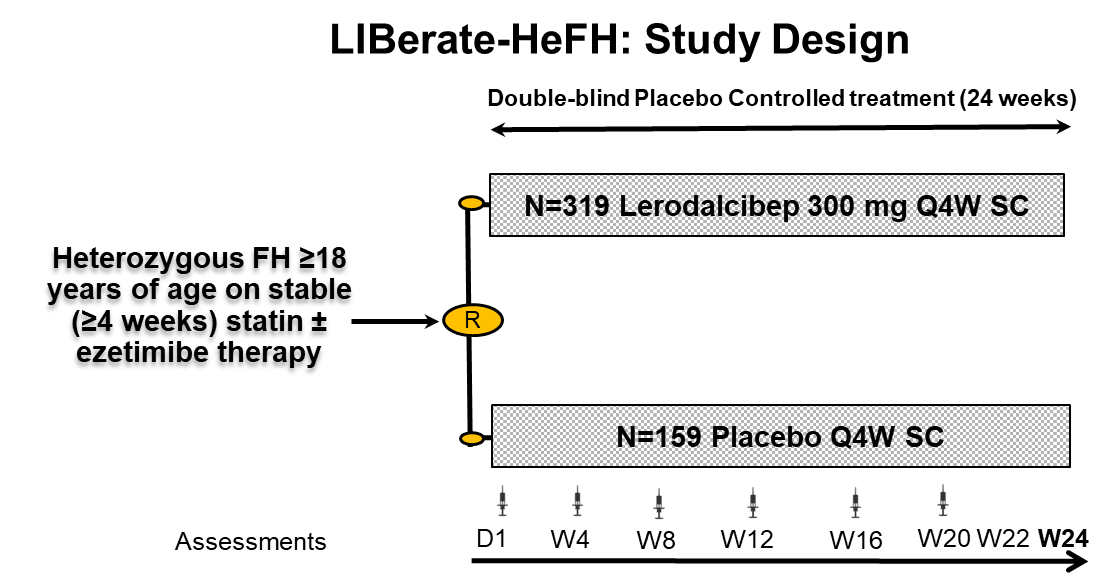  D = day; FH = familial hypercholesterolaemia; Q4W (≤31 days) = monthly; SC = subcutaneously; W = week.  Following randomization and dosing on Day 1, patients were seen in the clinic Q4W (≤31 days) for 20 weeks and then at 2-week intervals for Weeks 22 and 24. All patients received doses of lerodalcibep 300 mg Q4W (≤31 days) or matching placebo on Day 1 and Weeks 4, 8, 12, 16, and 20. In addition to a basic lipid profile, LDL-C was measured by preparative ultracentrifugation at specified visits during the study. An aliquot of the lipid specimen at Day 1 and Week 24/ET was stored for later analysis of exploratory lipid and CV risk biomarkers. A deoxyribonucleic acid sample was also obtained at the Randomization Visit for genetic confirmation and assessment of variants known to cause HeFH. All lipid, PK, and PCSK9 results from the baseline measurements following Day 1 were blinded to the Investigator and all site and Sponsor personnel involved in the study. Final safety assessments included adverse events and the results from physical examinations, electrocardiograms (ECGs), clinical laboratory tests (haematology, serum chemistry, and urinalysis), and immunogenicity testing. Injection site reactions (ISRs) were assessed at each visit. Safety, ISR, and lipid data were reviewed by a Data Safety Monitoring Board (DSMB) at pre-determined intervals throughout the program. If indicated by these assessments, ADAs were analyzed during study conduct to support safety monitoring. Patients with confirmed positive ADAs titers had neutralizing antibodies (NAbs), PK (LIB003), and free PCSK9 assessed and, if necessary, were further characterized for isotype, binding site, and affinity.  Patients completing the base study were eligible to enter an open-label extension (OLE) study under a separate protocol and informed consent, where they would receive lerodalcibep 300 mg Q4W (≤31 days) for an additional 72 weeks.  Patients on lerodalcibep who tested positive for binding, non-NAbs and have clinical sequelae that were considered safety-related at the final visit of this study (Week 24 or Early Termination [ET]) were asked to return for additional monthly follow-up testing if they terminated LIB003-004 and did not enter the OLE study.  Patients on lerodalcibep who tested positive for NAbs at the final visit of the study (Week 24/ET), and who did not enter the long-term extension study and who had no clinical sequelae, were asked to return for follow-up testing every 3 months until either NAbs were no longer detectable, considered stable or the patient had been followed for a period of at least 12 months. For patients who tested positive, but who had not received active drug, follow-up testing was not required. |
| --- |

## Independent Data Monitoring Committee

| **Name** | **Affiliation** | **Area of Expertise** |
| --- | --- | --- |
| Professor Alan Chait (Chair) | Professor Emeritus, University of Washington, Seattle | Cardiology and randomized clinical trials |
| Professor David Walters | Professor Emeritus, UCSF | Cardiology and randomized clinical trials |
| Professor Eva Lonn | Professor, McMaster University | Cardiology and randomized clinical trials |
| Dr Theresa Stern | BIA Clinical Group, Ann Arbor | Biostatistics, methodology and clinical trials |

# List of Investigators and Study Sites

| **Investigator Name** | **Study Site** |
| --- | --- |
| Traci Turner | Metabolic and Atherosclerosis Research Center, Cincinnati, Ohio, USA |
| Jean Bergeron | Clinique des Maladies Lipidiques de Quebec Inc. Quebec, Canada |
| Artuela Caku | Centre Hospitalier Universite de Sherbrooke (CHUS), Sherbrooke, Canada |
| Avishay Elis | Rabin Medical Center, Beilinson Hospital, Petah Tikva, Israel |
| Ronen Durst | Hadassah Hebrew University Medical Center - Cardiology - The Heart Institute, Jerusalem, Israel |
| Zafer Yalim | Afyon Universitesi - Kardiyoloji, Afyonkarahisar, Turkey |
| Meral Kayikcioglu | Ege University School of Medicine,Department of Cardiology, Izmir, Turkey |
| Bahadir Kirilmaz | Canakkale Onsekiz Mart University, Kepez, Turkey |
| Atac Celik | Tokat Gaziosmanpasa University, Faculty of Medicine Dept. of Cardiology, Tokat, Turkey |
| Irfan Duzen | Gaziantep University Sahinbey Research and Practice Hospital,Gaziantep, Turkey |
| Abdurraham Oguzhan | Erciyes University Faculty of Medicine, Melikgazi, Turkey |
| Ibrahim Basarici | Akdeniz University Medical Faculty Hospital, Antalya, Turkey |
| Frederick Raal | Wits Clinical Research (WCR) - Carbohydrate and Lipid Metabolism Research Unit, Johannesburg, South Africa |
| Dirk Blom | University of Cape Town - Lipid Laboratory, Cape Town, South Africa |
| Marc Abelson | Helderberg Research Institute, Somerset West, South Africa |
| Matthys Basson | Tiervlei Trial Centre (TTC), Cape Town, South Africa |
| Lesley Burgess | TREAD Research - Department of Cardiology, Parow, South Africa |
| Nyda Fourie | Iatros International, Bloemfontein, South Africa |
| Eli Heggen | Oslo University Hospital, Oslo, Norway |
| Emil Asprusten | The Lipid Clinic, Oslo University Hospital, Oslo, Norway |
| Vimal Mehta | GB Pant Institute of Postgraduate Medical Education & Research, New Dehli, India |
| Raman Puri | Indraprastha Apollo Hospitals, New Dehli, India |
| Ashwani Mehta | Sir Ganga Ram Hospital, New Dehli, India |
| Preeti Gupta | Vardhman Mahavir Medical College and Safdarjung Hospita, New Dehli, India |
| Milan Chag | Care Institute of Medical Science, Gujarat, India |
| Akshyaya Pradhan | King George's Medical University, Uttar Pradesh, India |
| Francisco Fuentes Jimenez | Hospital Universitario Reina Sofia, Cordoba, Spain |
| Fernando Civeira Murillo | Hospital Universitario Miguel Servet Zaragoza, Spain |
| Xavier Pinto Sala | Hospital Universitari de Bellvitge, Barcelona, Spain |
| Russell Scott | New Zealand Clinical Research, Christchurch, New Zealand |

# Trial Registration

Trial Registration number for the study is EudraCT: 2020-004390-44; Clinicaltrials.gov NCT04797104.

The trial was conducted between 3 May, 2021 (first patient screened) and 15 May, 2023 (last patient last visit)

# Study Methods


**3.1 Inclusion Criteria**

Patients had to meet all of the following criteria to be eligible to participate in the study:

Provision of written and signed informed consent prior to any study-specific procedure;

Male or female, ≥18 years of age at the first Screening Visit;

Weight of ≥40 kg (88 lbs) and body mass index (BMI) ≥17 and ≤42 kg/m^2^;

Diagnosis of definite, probable or possible HeFH based either on clinical criteria (Simon Broome criteria] or Dutch Lipid Clinics [DLC] Network Criteria or genotyping and at the defined eligibility visit (screening or post washout/stabilization) a calculated LDL-C (Friedewald) ≥ 1.8 mmol/L (70 mg/dL) (if ASCVD*) or ≥ 2.6 mmol/L (100 mg/dL) (if no ASCVD) and TG ≤ 4.5 mmol/L (400 mg/dL) while on stable lipid lowering oral drug therapy (e.g., statin with or without ezetimibe);

NOTE: ASCVD encompasses acute coronary syndrome, history of myocardial infarction (MI), stable or unstable angina or coronary or other arterial revascularization, stroke, transient ischemic attack, or peripheral arterial disease, including aortic aneurysm, all of atherosclerotic origin.

Patients unable to tolerate approved doses of a statin could take lower than approved doses and less frequently than daily as long as the dose and dosing frequency was consistent. Patients with documentation of inability to tolerate any statin at any dose, or history of rhabdomyolysis, and unable to tolerate *any other allowed oral lipid lowering agent*, and thus on no lipid lowering therapy had to have an LDL-C ≥4.9 mmol/L (190 mg/dL) at the Screening Visit unless they have a documented pathogenic FH variant;

On a stable diet and lipid lowering oral therapies (such as statins, ezetimibe, bile-acid sequestrants, OM-3 compounds, fenofibrate, bezafibrate or niacin) or combinations thereof for at least 4 weeks (excluded oral lipid lowering agents are defined below and included mipomersen, lomitapide, gemfibrozil, and bempedoic acid);

Patients on a PCSK9 mAb at a dose of 75 mg, 140 mg, or 150 mg Q2W had to undergo a washout period of ≥4 weeks after the last dose; for those on a dose of 300 mg or 420 mg Q4W (≤31 days) the washout period was ≥8 weeks following last dose; and

Females of childbearing potential had to be using a highly effective form of birth control^#^ if sexually active and have negative urine pregnancy test at the last Screening Visit;

NOTE: Highly effective methods of birth control include refraining from heterosexual sexual intercourse during the entire period of risk, birth control pills or patches, intrauterine devices (IUDs), sexual activity with a male partner who has had a vasectomy, condom or diaphragm or cervical cap with spermicide or IUD, oral, implantable, or injectable contraceptives. Menopause is defined as 12 months of spontaneous and continuous amenorrhea in a female <55 years old or 12 months of spontaneous and continuous amenorrhea with a follicle-stimulating hormone (FSH) level >40 IU/L (or according to the definition of “postmenopausal range” for the laboratory involved) in a female <55 years old unless the patient has undergone bilateral oophorectomy.

**3.2 Exclusion Criteria**

Patients who met any of the following criteria were excluded from participation in the study:

1. Use of prohibited oral lipid lowering agents mipomersen or lomitapide within 6 months of screening, gemfibrozil within 6 weeks of screening or bempedoic acid within 4 weeks of screening;
2. Low-density lipoprotein or plasma apheresis within 2 months prior to Day 1;
3. Documented history of homozygous familial hypercholesterolaemia defined as clinical and/or genetic with true HoFH (ie, identical pathogenic variants), compound heterozygous (ie, 2 different pathogenic LDLR variants) or combined heterozygous (2 different pathogenic FH variants such as LDLR plus Apo B, LDLR plus PCSK9 GOF);
4. History of any prior or active clinical condition or acute and/or unstable systemic disease compromising patient inclusion, at the discretion of the Investigator, including but not limited to clinically significant pulmonary, hematologic, gastrointestinal, endocrine (excluding diabetes), immunologic, dermatologic, neurologic, or psychiatric disease, which in the Investigator’s opinion would not be suitable for the study from a patient safety consideration or could interfere with the results of the study;
5. Females of childbearing potential who are sexually active, not using or unwilling to use a highly effective form of contraception#, pregnant or breastfeeding, or who have a positive urine pregnancy test at the last Screening Visit;

*#Highly effective methods of birth control include refraining from heterosexual sexual intercourse during the entire period of risk, birth control pills or patches, IUDs, sexual activity with a male partner who has had a vasectomy, condom or diaphragm or cervical cap with spermicide or IUD, oral, implantable, or injectable contraceptives.*

1. Moderate to severe renal dysfunction, defined as an estimated glomerular filtration rate <30 mL/min/1.73m^2^ at the Screening Visit;
2. Active liver disease or hepatic dysfunction (eg, cirrhosis, alcoholic liver disease, known hepatitis B [HBV] or hepatitis C [HCV], autoimmune hepatitis, liver failure, liver cancer), history of liver transplant, and/or AST or ALT >2.5 × the ULN as determined by central laboratory analysis at screening (tests that result in ALT or AST up to 3 × ULN may have 1 repeat test to confirm eligibility during the Screening Period);
3. Uncontrolled thyroid disease: hyperthyroidism or hypothyroidism as defined by thyroid stimulating hormone (TSH) below the lower limit of normal (LLN) or >1.5 × ULN, respectively, at the Screening Visit. If TSH is above/below these cut-off points, the subject can enter if the free T3 is within the reference range. If controlled, then treatment should be stable for at least 3 months prior to the Screening Visit;
4. Uncontrolled Type 1 or Type 2 diabetes mellitus (defined as fasting glucose ≥200 mg/dL or glycated hemoglobin (HbA1c) of ≥9%);
5. Uncontrolled serious cardiac arrhythmia (sustained ventricular tachycardia, frequent non‑sustained ventricular tachycardia, any ventricular fibrillation episode, wide-complex tachycardia, atrial fibrillation with rapid ventricular response, and severe second degree or third degree atrioventricular block), MI, unstable angina, percutaneous coronary intervention, coronary artery bypass grafting, placement of implantable cardioverter defibrillator or biventricular pacemaker, aortic valve surgery, or stroke within 3 months prior to enrollment (the day patient signed the informed consent and first procedure was performed);
6. Planned cardiac surgery or revascularization;
7. New York Heart Association III-IV heart failure; or patients with last documented left ventricular ejection fraction <30% by SOC assessments, eg, echocardiography, cardiac magnetic resonance imaging, nuclear imaging, computed tomography angiography, angiography with ventriculogram, within 12 months;
8. Uncontrolled hypertension defined as evidenced by a reproducible (repeated 5 minutes apart) sitting blood pressure >160 mmHg systolic or >100 mmHg diastolic;
9. Enrolled in another investigational device or drug study, or less than 30 days or 5 half-lives since ending another investigational device or drug study(ies), or receiving other investigational agent(s); such as PCSK9 or Lp(a) siRNA or locked nucleic acid‑reducing agents within 12 months of the Screening Visit;
10. Unexplained creatine kinase >5 × ULN, unless related to exercise or unusual activity in which case 1 repeat test is allowed;
11. Patients who could not be available for protocol-required study visits or procedures, to the best of the patient’s and Investigator’s knowledge;
12. A history, within 6 months prior to screening, of prescription drug abuse, illicit drug use, or alcohol abuse according to medical history;
13. Donated or lost a significant volume (>500 mL) of blood/plasma within 30 days prior to Day 1;
14. Had a blood transfusion within 4 weeks of randomization or known diagnosis of human immunodeficiency virus;
15. Were previously treated with lerodalcibep or any adnectin product or have a history of allergy to evolocumab;
16. Have any other finding which, in the opinion of the Investigator, would compromise the patient’s safety or participation in the study; or
17. An employee or family member of the Investigator or study site personnel.

## 3.3 Baseline Statin Dose Categories

| **High-intensity Statins** | **Moderate-intensity Statins** | **Low-intensity Statins*** |
| --- | --- | --- |
| Atorvastatin 40 – 80 mg | Atorvastatin 10 – 20 mg | Simvastatin 10 mg |
| Rosuvastatin 20 – 40 mg | Rosuvastatin 5 – 10 mg | Pravastatin 10 – 20 mg |
| Simvastatin 80mg | Simvastatin 20 – 40 mg | Lovastatin 20 mg |
|  | Pravastatin 40 – 80 mg | Fluvastatin 20 – 40 mg |
|  | Lovastatin 40 mg | Pitavastatin 1 mg |
|  | Fluvastatin XL 80 mg |  |
|  | Fluvastatin 40 mg twice daily |  |
|  | Pitavastatin 2 – 4 mg |  |
| *Low-intensity statins also include those patients taking low-dose statins using an alternate regimen (i.e., every other day or for a specified number of times per week). | | |

## 3.4 Laboratory Analytical Methods

All analyses were performed by Medpace Reference laboratories (MRL) who are certified in the CDC-NHLBI Lipid Standardization Part III Program.

PCSK9 analysis was performed using Quantikine ELISA from R&D Systems according to the manufacturer’s instructions using a Tecan Sunrise reader and EDTA-plasma. Intra- and inter-assay coefficients of variation were observed to be 4.7% and 5.4%. Completed subject sets were analyzed on the same plate to eliminate inter-assay variability when comparing results within a subject.

LDL cholesterol was determined by both the Friedewald formula, and additionally by preparative ultracentrifugation (PUC). LDL cholesterol methods remained the same throughout the trial, including both calibration and reagent systems. MRL does not change methods during the course of a study.

Analytical methods (includes all methods required to derive LDL cholesterol by Friedewald and PUC)

Analysis of total cholesterol (TC) and triglycerides (TG) were by enzymatic methods on a Beckman Coulter AU Series automatic analyzer with in-house developed serum calibrators directly traceable to CDC-NHLBI reference procedures. (Ref: Myers GL, Cooper GR, et al. The Centers for Disease Control-National Heart, Lung and Blood Institute Lipid Standardization Program. An approach to accurate and precise lipid measurements. *Clin Lab Med* 1989;9:105-35).

HDL cholesterol was performed by precipitation with 50 kDa dextran sulfate with magnesium ions (MgCl2), followed by analysis of the supernatant for cholesterol by enzymatic methods on a Beckman Coulter AU Series automatic analyzer with in-house developed serum calibrators directly traceable to CDC-NHLBI reference procedures (same methodology as TC).

PUC was performed using the method outlined in the Lipid Research Clinics methods manual. (Ref: US Department of Health and Human Services. Manual of laboratory operations: lipid and lipoprotein analysis (revised). Washington, DC: US Government Printing Office; 1982. Report No.: (NIH) 75-67815).

Serum or plasma was overlaid with normal saline (density 1.006 g/mL) and centrifuged (Beckman Ultracentrifuge Model # L-90K and rotor, Type 50.4) at 40,000 rpm for 18–22 hours at 10°C to separate very low-density lipoprotein (VLDL) in the supernatant (‘top’ fraction) from LDL and HDL in the infranatant or ‘bottom’ fraction. The cholesterol concentration of the infranatant was measured. All apolipoprotein B-containing lipoproteins, VLDL, LDL and Lp(a), were precipitated from serum using 50 kDa dextran sulfate with magnesium ions (MgCl2), and the cholesterol in the remaining HDL fraction was measured. The HDL cholesterol concentration was subtracted from the infranatant cholesterol to provide the PUC LDL cholesterol value.

Calculated LDL cholesterol was derived from the Friedewald formula where:

LDL cholesterol (mg/dL) = TC – (HDL cholesterol + TG/5)

[for mmol/L, LDL cholesterol = TC – HDL cholesterol – (TG/2.2)].

Lp(a): MRL uses an automated monoclonal antibody immunoturbidometric “isoform-independent” method, meaning independent of Lp(a) particle size. The calibrators for this kit are referenced against WHO SRM 2B. Since there is significant heterogeneity in Lp(a) particles, the assay used should be insensitive to the size of apo(a)─the protein bound to apo B-100. Results are reported in nmol/L of Lp(a) protein, rather than mass units, due to the varying mass ratio of apo(a) to apoB in different sized Lp(a) particles. The Polymedco Lp(a) method in use at MRL is analyzed on a Beckman Coulter AU Series analyzer, and is referenced to an International Reference Material (SRM 2B) developed by the International Federation of Clinical Chemistry (IFCC), and approved by National Heart, Lung, and Blood Institute (NHLBI). This allows accurate measurement of Lp(a) levels irrespective of isoform size.

Apolipoprotein B was measured using nephelometric methodology on a Siemens BNII analyzer.

## 3.5 Sample Size

The number of patients was based on administration of Lerodalcibep 300 mg Q4W (≤31 days) achieving at least 95% power to detect a statistically significant difference in LDL-C level at both Week 24 and at the mean of Weeks 22 and 24 compared to the placebo group at 2-sided alpha level of 0.05, assuming a common standard deviation of 17.5% for percent change in LDL-C and a treatment difference of 50% as determined by literature review. In terms of safety, while the number of patients was not based on statistical consideration, there was an 80% probability of observing at least 1 occurrence in the Lerodalcibep treatment group of any adverse event which would occur with a 24% incidence in the population from which the sample is drawn. Approximately 600 patients were required to participate in this clinical study, including 400 to receive lerodalcibep and 200 to receive placebo.

## 3.6 Statistical Analyses – Efficacy End Points

The coprimary efficacy endpoints were to assess percent change from baseline compared to placebo in LDL-C level at Week 24 (by Friedewald formula) and LDL-C level at the mean of Weeks 22 and 24 (by Friedewald formula). Percent change from baseline in LDL-C was analyzed with analysis of covariance model with treatment and the CVD status as factors and baseline value as a covariate. For patients in the Intent-to-Treat (ITT) Population who withdrew early, a multiple imputation was used to explore the potential impact of missing data. Other efficacy variables were analyzed similarly. For analysis of each primary objective, the treatment comparison used a significance level of 0.05.

Other efficacy variables were analyzed similarly. The percentage of patients achieving a ≥ 50% reduction in LDL-C and in those with ASCVD an LDL-C < 1.4 mmol/L and the percentage of patients without ASCVD achieving a LDL-C < 1.8 mmol/L was analyzed using logistic regression model with treatment and CVD status as factors and baseline value as a covariate. Multiplicity adjustment was based on a combination of sequential testing, the Hochberg procedure, and fallback procedure to control the overall signiﬁcance level for the primary and key secondary endpoints. A sensitivity analysis of the coprimary endpoints was also performed by application of a mixed-eﬀect model repeat measurement and rank analysis of covariance (Quade test) for patients who adhered to the scheduled study drug administration with visits < 31 days and who did not have any missing data for the coprimary endpoints.

# Supplementary Figures

**Figure S1. Consort Diagram: Participant Disposition**


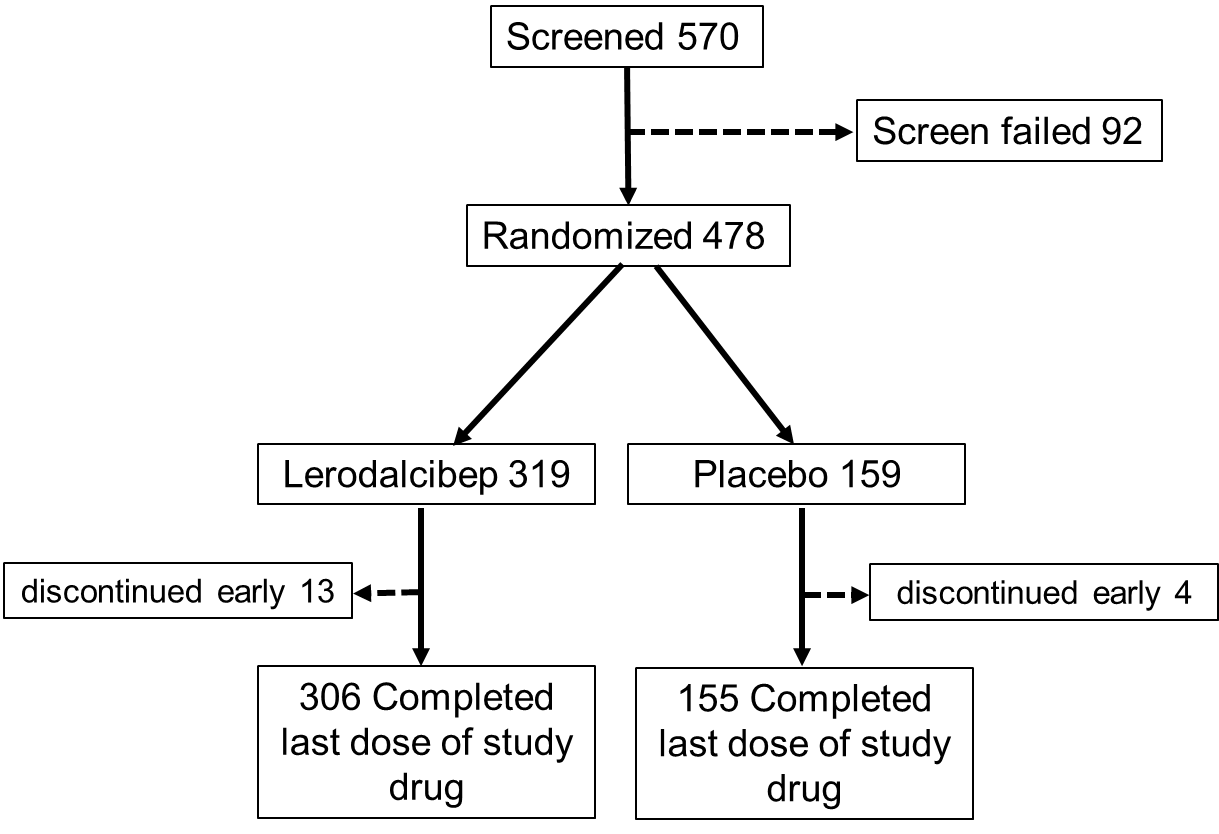


**Figure S2**.

**Waterfall plot of percent change in LDL-cholesterol (Friedewald formula) from baseline to Week 24 with lerodalcibep and placebo in individual patients**


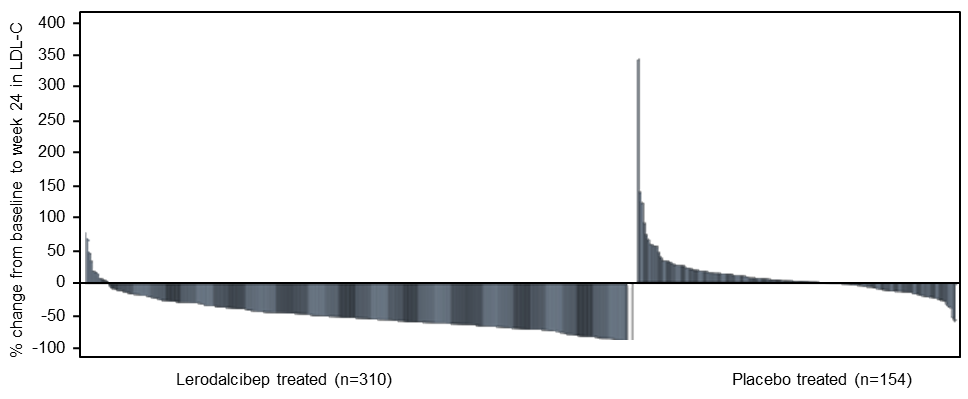


# Supplementary Tables

**Table S1. Injection site reactions (ISRs) – safety population**

| **Treatment** | **Lerodalcibep (N=318)** | **Placebo (N=159)** |
| --- | --- | --- |
| Patients with ISRs N(%) | 32 (10.1%) | 2 (1.3%) |
| ISR grade: Mild | 28 | 1 |
| Moderate | 4 | 1 |
| Severe | 0 | 0 |
| ISR type: Erythema | 21 | 1 |
| Bruising | 6 | 1 |
| Pruritus | 4 | 0 |
| Pain | 1 | 0 |
| Total doses administered: | 1853 | 934 |
| ISR events N(%) | 57(3.1%) | 2(0.2%) |

**Table S2. Percent Change from Baseline LDL-C (Friedewald) at W24 and the Mean of W22/24: Per-Protocol Population**

**a) Week 24**

|  | Placebo | Lerodalcibep |
| --- | --- | --- |
| N | 130 | 269 |
| LDL-C (mmol/L) |  |  |
| baseline: mean(SD) | 3.81 (1.48) | 3.84 (1.69) |
| Week 24: mean(SD) | 3.95 (1.65) | 1.73 (1.30) |
| % change |  |  |
| LS mean (SE) | 7.45 (2.73) | -56.17 (1.92) |
| 95% CI | 2.09, 12.81 | -59.94, -52.41 |
| Comparison vs PBO |  |  |
| LS mean (SE) |  | **-63.62 (3.27)** |
| 95% CI |  | -70.05, -57.19 |
| p value |  | <0.0001 |

**b) Mean of Week 22 and week 24**

|  | Placebo | LIB003 |
| --- | --- | --- |
| N | 130 | 269 |
| LDL-C (mmol/L) |  |  |
| baseline: mean(SD) | 3.81 (1.48) | 3.84 (1.69) |
| W 22/24: mean(SD) | 3.90 (1.59) | 1.48 (1.24) |
| % change |  |  |
| LS mean (SE) | 6.22 (2.36) | -64.01 (1.66) |
| 95% CI | 1.58, 10.85 | -67.27, -60.76 |
| Comparison vs PBO |  |  |
| LS mean (SE) |  | **-70.23 (2.83)** |
| 95% CI |  | -75.79, -64.67 |
| p value |  | <0.0001 |

**Table S3: Patients Achieving Current ESC/EAS Guidelines**

**a) Any Trough Visit - ITT population**

| **Treatment** | **Lerodalcibep** | **Placebo** | **Odds Ratio** | **p-value** |
| --- | --- | --- | --- | --- |
| All patients: | N=319 | N= 159 |  |  |
| ≥50% LDL-C reduction | 86.2% | 3.8% | 186 | <0.0001 |
| Achieved LDL-C target | 69.6% | 4.4% | 122 | <0.0001 |
| Both goals | 68.0% | 1.9% | 220 | <0.0001 |
| ASCVD/very-high risk ASCVD | N=203 | N=102 |  |  |
| ≥50% LDL-C reduction | 85.7% | 3.9% | 178 | <0.0001 |
| Achieved LDL-C target | 68.0% | 5.9% | 85 | <0.0001 |
| Both goals | 67.0% | 2.0% | 218 | <0.0001 |
| High-risk for ASCVD | N=116 | N=57 |  |  |
| ≥50% LDL-C reduction | 87.1% | 3.5% | 202 | <0.0001 |
| Achieved LDL-C target | 72.4% | 1.8% | 334 | <0.0001 |
| Both goals | 69.8% | 1.8% | 229 | <0.0001 |

LDL-C = LDL-cholesterol by Friedewald formula

Targets: <1.4 mmol/L if ASCVD/very-high risk for ASCVD or <1.8 mmol/L if high-risk for ASCVD

**b) Mean of Weeks 22/24 Visits - ITT population**

| **Treatment** | **Lerodalcibep** | **Placebo** | **Odds Ratio** | **p-value** |
| --- | --- | --- | --- | --- |
| All patients: | N=319 | N= 159 |  |  |
| ≥50% LDL-C reduction | 74.3% | 0.6% | 637 | <0.0001 |
| Achieved LDL-C target | 60.5% | 0.6% | 627 | <0.0001 |
| Both goals | 58.6% | 0.0% | 918 | <0.0001 |
| ASCVD/very-high risk ASCVD | N=203 | N=102 |  |  |
| ≥50% LDL-C reduction | 76.80% | 1.0% | 501 | <0.0001 |
| Achieved LDL-C target | 60.10% | 1.0% | 467 | <0.0001 |
| Both goals | 59.10% | 0.0% | 756 | <0.0001 |
| High-risk for ASCVD | N=116 | N=57 |  |  |
| ≥50% LDL-C reduction | 69.80% | 0.0% | 305 | <0.0001 |
| Achieved LDL-C target | 61.20% | 0.0% | 349 | <0.0001 |
| Both goals | 57.80% | 0.0% | 241 | <0.0001 |

LDL-C = LDL-cholesterol by Friedewald formula

Targets: <1.4 mmol/L if ASCVD/very-high risk for ASCVD or <1.8 mmol/L if high-risk for ASCVD
